# Mobile Health Applications for Secondary Prevention After Myocardial Infarction or PCI: A Systematic Review and Meta-Analysis of Randomized Controlled Trials

**DOI:** 10.3390/healthcare13151881

**Published:** 2025-08-01

**Authors:** Ioannis Skalidis, Henri Lu, Niccolo Maurizi, Stephane Fournier, Grigorios Tsigkas, Anastasios Apostolos, Stephane Cook, Juan F. Iglesias, Philippe Garot, Thomas Hovasse, Antoinette Neylon, Thierry Unterseeh, Jerome Garot, Nicolas Amabile, Neila Sayah, Francesca Sanguineti, Mariama Akodad, Panagiotis Antiochos

**Affiliations:** 1 Institut Cardiovasculaire Paris-Sud, Hôpital Jacques Cartier, Ramsay-Santé, 91300 Massy, France; 2 School of Medicine, University of Crete, 71500 Heraklion, Greece; 3 Service of Cardiology, Lausanne University Hospital, 1011 Lausanne, Switzerland; 4 Department of Cardiology, University Hospital of Patras, 26504 Patras, Greece; 5 First Department of Cardiology, Hippokration General Hospital, 11527 Athens, Greece; 6 Department of Cardiology, University and Hospital Fribourg, 1700 Fribourg, Switzerland; 7 Division of Cardiology, University Hospital Geneva, 1211 Geneva, Switzerland

**Keywords:** coronary artery disease, digital health, meta-analysis, mHealth, percutaneous coronary intervention (PCI), secondary prevention

## Abstract

**Background:** Mobile health applications have emerged as a novel tool to support secondary prevention after myocardial infarction (MI) or percutaneous coronary intervention (PCI). However, the impact of app-based interventions on clinically meaningful outcomes such as hospital readmissions remains uncertain. **Objective:** To systematically evaluate the effectiveness of smartphone app-based interventions in reducing unplanned hospital readmissions among post-MI/PCI patients. **Methods:** A systematic search of PubMed was conducted for randomized controlled trials published between January 2020 and April 2025. Eligible studies evaluated smartphone apps designed for secondary cardiovascular prevention and reported on unplanned hospital readmissions. Risk ratios (RRs) and 95% confidence intervals (CIs) were pooled using a random-effects model. Subgroup analyses were performed based on follow-up duration and user adherence. **Results:** Four trials encompassing 827 patients met inclusion criteria. App-based interventions were associated with a significant reduction in unplanned hospital readmissions compared to standard care (RR 0.45; 95% CI: 0.23–0.89; *p* = 0.0219). Greater benefits were observed in studies with longer follow-up durations and higher adherence rates. Improvements in patient-reported outcomes, including health-related quality of life, were also documented. Heterogeneity was moderate. Major adverse cardiovascular events (MACEs) were reported in only two studies and were not analyzed due to inconsistent definitions and low event rates. **Conclusions:** Smartphone applications for post-MI/PCI care are associated with reduced unplanned hospital readmissions and improved patient-reported outcomes. These tools may play a meaningful role in future cardiovascular care models, especially when sustained engagement and personalized features are prioritized.

## 1. Introduction

Cardiovascular diseases remain the leading cause of morbidity and mortality globally. Patients with a history of myocardial infarction (MI) and/or recent percutaneous coronary intervention (PCI) are key populations requiring structured secondary prevention. While pharmacologic management and cardiac rehabilitation are cornerstones of care, patient adherence and follow-up engagement remain inadequate in many settings, contributing to recurrent events and rehospitalizations [1,2].

Digital health technologies have gained prominence as potential solutions to support long-term cardiovascular care. Among these, smartphone applications (apps) provide a unique platform to deliver continuous patient engagement, real-time monitoring, and tailored behavioral interventions. For example, a smartphone-based digital health program implemented during cardiac rehabilitation demonstrated a non-significant 28% relative reduction in the combined rate of rehospitalizations and emergency department visits at 180 days post-PCI [3]. Similarly, the eMOTIVA app significantly improved adherence to the Mediterranean diet, physical activity levels, sedentary behavior, and exercise capacity at 6 months in patients following PCI [4]. These apps can support guideline-based management through educational content, physical activity tracking, medication reminders, and remote symptom reporting. The COVID-19 pandemic has further accelerated the adoption of mobile health (mHealth) strategies, underscoring their relevance in decentralized care.

Despite widespread development and implementation, the clinical impact of app-based interventions on hard outcomes such as unplanned hospital readmissions remain unclear. Previous reviews have grouped heterogeneous digital tools, including websites, SMS platforms, or wearable devices, diluting the specific effects of smartphone apps. Moreover, differences in app functionality, study design, and outcome definitions pose challenges in interpreting the literature [5,6].

This systematic review and meta-analysis aims to provide a focused, quantitative synthesis of RCTs evaluating app-based interventions for secondary prevention in patients with recent MI or PCI. The primary objective is to determine whether these interventions reduce hospital readmissions and urgent cardiac care utilization. Secondary objectives include evaluating heterogeneity by intervention duration, geographic setting, and patient adherence.

## 2. Methods

### 2.1. Study Design and Protocol Development

This systematic review and meta-analysis was designed in accordance with the PRISMA 2020 guidelines. A protocol was developed to predefine the study rationale, research questions, eligibility criteria, search strategy, and statistical approach. Although not prospectively registered, the protocol was followed rigorously throughout the study.

### 2.2. Search Strategy

We conducted a comprehensive literature search in the PubMed database to identify relevant randomized controlled trials published between 1 January 2020, and 30 April 2025. The search strategy employed Boolean logic with a combination of Medical Subject Headings (MeSHs) and free-text terms. These included “myocardial infarction”, “acute coronary syndrome”, “percutaneous coronary intervention”, “secondary prevention”, “digital health”, “mobile health”, “smartphone application”, “mHealth”, and “eHealth.” Filters were applied to restrict results to human subjects, adults aged 18 and older, and randomized controlled trials. In addition to the primary search, we performed backward citation screening of included studies and relevant reviews to identify any additional eligible trials.

### 2.3. Eligibility Criteria

We applied the PICOS framework to define inclusion and exclusion criteria. Eligible studies were randomized controlled trials enrolling adult patients with a confirmed history of myocardial infarction or recent PCI. The intervention had to consist of a smartphone application delivering components of secondary prevention, such as educational content, lifestyle modification support, medication reminders, remote monitoring, or structured follow-up. The comparator could include standard care, printed materials, or minimal intervention without digital health components. Studies were required to report at least one clinically relevant outcome, including unplanned hospital readmission, urgent cardiac visits, or major adverse cardiovascular events (MACEs). We excluded non-randomized studies, observational research, pilot feasibility trials without clinical endpoints, and interventions lacking a smartphone app as the primary platform.

### 2.4. Study Selection and Screening Process

Two independent reviewers conducted the screening process in two stages. First, titles and abstracts of all identified records were evaluated for relevance. Articles that appeared potentially eligible were retrieved for full-text review. Disagreements at any stage were resolved by consensus or by consulting a third reviewer. A standardized form was used to document the inclusion decision and reason for exclusion where applicable. Inter-reviewer agreement was assessed using Cohen’s kappa statistic to quantify consistency.

### 2.5. Data Extraction and Outcomes of Interest

We developed a structured data extraction form to systematically collect relevant information. This included general study characteristics (author, year, journal, country), patient demographics (sample size, age, sex, comorbidities), details of the intervention (app name, features, duration, frequency of use), comparator information, outcomes measured, length of follow-up, and engagement or adherence data. The primary outcome was a composite of all-cause unplanned hospital readmissions or urgent cardiac care visits. Secondary outcomes included major adverse cardiovascular events (MACEs), typically comprising cardiovascular death, recurrent myocardial infarction, or non-fatal stroke. We also extracted data on app engagement and adherence metrics when available, such as logins, feature use, module completion, or percentage of patients actively using the app at follow-up. Data were extracted by two reviewers independently, and discrepancies were resolved through consensus.

### 2.6. Risk of Bias Assessment and Statistical Analysis

Each included study was independently assessed for risk of bias using the Cochrane Risk of Bias 2.0 tool, which evaluates five domains: the randomization process, deviations from intended interventions, missing outcome data, measurement of outcomes, and selective reporting. Studies were categorized as having low risk, some concerns, or high risk of bias. For statistical synthesis, we applied a random-effects meta-analysis model using the DerSimonian and Laird method to account for between-study heterogeneity. Risk ratios (RRs) and corresponding 95% confidence intervals (CIs) were calculated for dichotomous outcomes. All included trials reported outcomes as event counts over defined follow-up periods rather than time-to-event estimates. Accordingly, hazard ratios (HRs) were not available or applicable, and RRs with 95% confidence intervals were calculated directly from extracted event and sample size data using the Mantel–Haenszel method. Heterogeneity across studies was assessed using the I^2^ statistic, with thresholds of 25%, 50%, and 75% representing low, moderate, and high heterogeneity, respectively. Subgroup analyses were conducted based on duration of follow-up (≤6 months vs. >6 months), country income classification (high income vs. upper-middle income), and reported adherence to the intervention (≥75% vs. <75%). Sensitivity analyses were performed excluding studies at high risk of bias. Due to the small number of included studies, formal assessment of publication bias (e.g., funnel plot asymmetry) was not performed. All meta-analyses were performed using Review Manager (RevMan) version 5.4.

## 3. Results

### 3.1. Study Selection

**Figure 1:**
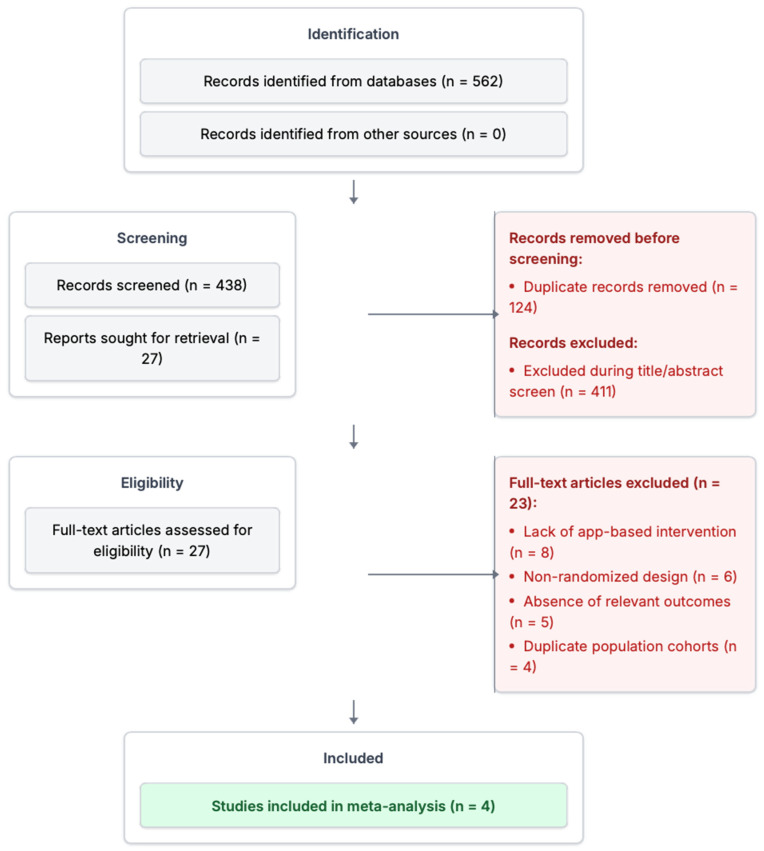
PRISMA flowchart. Flow diagram outlining the study selection process for inclusion in the systematic review and meta-analysis. PRISMA, Preferred Reporting Items for Systematic Reviews and Meta-Analyses.

A total of 562 records were identified through database searches. No additional records were found through other sources. Following the removal of 124 duplicate records, 438 titles and abstracts were screened. Of these, 411 were excluded during the title and abstract screening for not meeting the inclusion criteria. The remaining 27 articles were retrieved for full-text assessment. After a detailed review, 23 articles were excluded for the following reasons: lack of app-based intervention (n = 8), non-randomized study design (n = 6), absence of relevant outcomes (n = 5), and duplicate population cohorts (n = 4). Ultimately, four studies met all inclusion criteria and were included in the meta-analysis. The study selection process is illustrated in the PRISMA flow diagram (Figure 1).

### 3.2. Study Characteristics

The four included randomized controlled trials were conducted in diverse geographical regions: TELE-ACS in the United Kingdom, afterAMI in Poland, ToDo-CR in Australia, and WeChat HBCR in China [7,8,9,10]. All were published between 2022 and 2023. Sample sizes ranged from 100 to 337 participants, with a total pooled population of 827 patients. Interventions were delivered via smartphone applications that included features such as educational content, medication reminders, remote symptom tracking, bidirectional clinician communication, and behavior modification tools. Follow-up durations ranged from 3 to 42 months. Control groups received usual care without structured digital health support. The primary objectives varied but commonly included reducing hospital readmissions, improving adherence to secondary prevention strategies, and enhancing patient-reported outcomes. TELE-ACS focused on individualized monitoring and symptom tracking, ToDo-CR emphasized physical activity and behavioral activation, and WeChat HBCR integrated structured cardiac rehabilitation tailored to the local healthcare system. Comparators across studies uniformly involved usual care without app-based support. In TELE-ACS and afterAMI, this entailed standard post-discharge cardiology follow-up. In ToDo-CR, control patients received printed materials and center-based rehabilitation invitations. In WeChat HBCR, control participants had access to conventional outpatient follow-up and unstructured education, without remote monitoring (Table 1).

### 3.3. Population Characteristics

Participants were adult patients with a recent myocardial infarction or percutaneous coronary intervention (PCI). Inclusion typically occurred within 2 to 12 weeks after index MI or PCI. The proportion of patients with recent acute MI ranged from 56% to 72%, with the remainder being post-elective PCI for stable CAD. The mean age across studies ranged from 56.8 to 67.4 years, with a pooled average of 61.7 years. Men comprised approximately 66.5% of participants. Common cardiovascular risk factors included hypertension (49–76%), hyperlipidemia (58–85%), diabetes mellitus (12–36%), and current tobacco use (17–42%). A prior history of myocardial infarction or coronary revascularization (either PCI or CABG) was present in 20–30% of participants across the included trials. Baseline medication use was consistently high, with over 90% of patients receiving dual antiplatelet therapy and statins. Ethnic composition varied by region: European trials primarily enrolled White patients, while the Chinese study involved exclusively Han Chinese participants. Socioeconomic status and digital literacy were not uniformly reported and may have influenced engagement levels (Table 2). Heart failure with reduced or preserved ejection fraction (HFrEF or HFpEF) was not consistently reported across the included studies, precluding subgroup analysis for this clinically important population.

### 3.4. Intervention Features and App Adherence

All interventions utilized native smartphone applications compatible with Android and iOS systems. Common functionalities included educational content on cardiovascular health, interactive modules on physical activity and diet, medication reminders, symptom-tracking tools, and mechanisms for communication with clinicians or case managers. The TELE-ACS app featured personalized care plans and weekly check-ins, while afterAMI included daily push notifications and secure messaging. ToDo-CR emphasized behavioral reinforcement and included wearable integration for step count tracking. The WeChat HBCR program offered group-based peer support and real-time physician feedback. Adherence data were reported in three of the four studies. TELE-ACS reported sustained app use in 81% of participants across 12 months. In afterAMI, 76% of patients interacted with the app at least once per week over the 6-month period. WeChat HBCR showed the highest adherence, with over 90% of participants completing ≥75% of recommended activities. Higher adherence rates were correlated with better clinical outcomes, particularly reductions in healthcare utilization and improved control of cardiovascular risk factors. However, intermediate adherence trends—such as changes at 1, 3, or 6 months—were not consistently reported, limiting insight into the temporal dynamics of engagement.

### 3.5. Primary Outcome: Unplanned Hospital Readmissions

**Figure 2:**
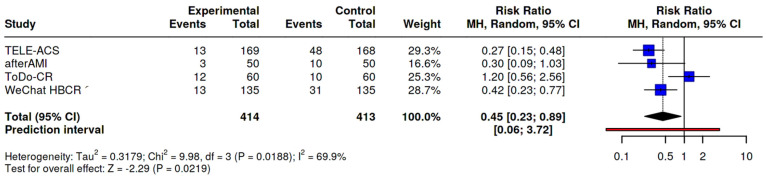
Forest plot—primary outcome: unplanned hospital readmissions.

All four included studies reported data on all-cause unplanned hospital readmissions. In the pooled analysis, app-based interventions were associated with a significant reduction in readmission risk compared to control (risk ratio [RR] 0.45; 95% confidence interval [CI] 0.23–0.89; *p* = 0.0219), indicating a 55% relative risk reduction. TELE-ACS and WeChat HBCR showed individually significant effects, while afterAMI and ToDo-CR did not reach statistical significance on their own. Moderate heterogeneity was observed (I^2^ = 69.9%), likely reflecting variability in follow-up duration, healthcare context, and intervention design. The wide prediction interval (0.06–3.72) suggests that effect sizes may differ across settings or populations (Figure 2).

Risk ratios (RRs) with 95% confidence intervals (CIs) were calculated using a Mantel–Haenszel random-effects model. Data from four randomized controlled trials (TELE-ACS, afterAMI, WeChat HBCR, and ToDo-CR) were included. Each study reported the number of participants with at least one unplanned hospital readmission over follow-up periods ranging from 6 to 42 months. Overall, digital interventions were associated with a significantly reduced risk of unplanned hospital readmission (RR 0.45, 95% CI 0.23 to 0.89, *p* = 0.0219). Heterogeneity was moderate (I^2^ = 69.9%). The prediction interval suggests variability in treatment effects across settings (0.06 to 3.72). 

The absolute event rate for unplanned readmissions was 9.9% in the intervention groups and 24.0% in control groups (median follow-up: 9 months), yielding an absolute risk reduction (ARR) of 14.1%. This corresponds to an estimated number needed to treat (NNT) of 7 to prevent one hospital readmission. As this figure is derived from pooled raw event rates and not from time-to-event analyses, it should be interpreted as an approximate clinical estimate (Table 3).

### 3.6. Secondary Outcomes: Patient-Reported Outcomes

Three of the four included trials (TELE-ACS, afterAMI, and WeChat HBCR) reported patient-reported outcomes. TELE-ACS and afterAMI demonstrated statistically significant improvements in health-related quality of life (HRQoL) using validated tools such as EQ-5D and SF-12. WeChat HBCR reported enhanced patient satisfaction and engagement with cardiac rehabilitation modules, though formal QoL scales were not used. ToDo-CR did not report QoL outcomes. Two studies (afterAMI and WeChat HBCR) also reported major adverse cardiovascular events (MACEs), but the definitions and follow-up durations differed. Due to this heterogeneity and low event numbers, MACEs were not pooled in the meta-analysis.

### 3.7. Subgroup and Sensitivity Analyses

Subgroup analysis based on follow-up duration showed that studies with longer follow-up (>6 months: TELE-ACS and WeChat HBCR) had a pooled risk ratio (RR) of 0.37 (95% CI: 0.17–0.79; I^2^ = 0%), while studies with ≤6 months follow-up (afterAMI and ToDo-CR) had a pooled RR of 0.74 (95% CI: 0.26–2.06; I^2^ = 71%). Geographically, stratification by country income classification indicated that high-income country trials (TELE-ACS, afterAMI, ToDo-CR) showed a pooled RR of 0.54 (95% CI: 0.26–1.13), and the upper-middle-income trial (WeChat HBCR) reported an individual RR of 0.42 (95% CI: 0.22–0.79). Trials with high adherence (≥75% of participants) to the app (TELE-ACS, afterAMI, WeChat HBCR) showed a pooled RR of 0.39 (95% CI: 0.20–0.78; I^2^ = 26%). The one study with unreported adherence (ToDo-CR) had a nonsignificant effect (RR 1.20; 95% CI: 0.53–2.74). Sensitivity analysis excluding ToDo-CR did not materially change the pooled effect (RR 0.39; 95% CI: 0.21–0.71; I^2^ = 0%).

## 4. Discussion

This systematic review and meta-analysis demonstrates that mobile health applications significantly reduce the risk of unplanned hospital readmissions in patients with recent MI/PCI. Across four randomized controlled trials enrolling 827 participants, app-based interventions were associated with a pooled RR of 0.45 (95% CI: 0.23–0.89; *p* = 0.0219), indicating a 55% relative reduction in readmissions compared to standard care. The absolute risk reduction of 14.1% corresponds to a NNT of 7, reflecting a meaningful clinical benefit. These findings are particularly noteworthy given the heterogeneity of digital health strategies employed, suggesting that even across diverse platforms and healthcare settings, mobile interventions can effectively bridge the transitional care gap and reduce preventable acute care utilization. The results also affirm the hypothesis that structured digital engagement post-discharge improves outcomes by enhancing adherence, symptom recognition, and patient–provider communication—mechanisms known to influence rehospitalization risk.

Subgroup analyses further delineated the conditions under which digital interventions are most effective. Trials with extended follow-up durations (>6 months) yielded greater reductions in readmission risk (RR 0.37; 95% CI: 0.17–0.79; I^2^ = 0%) compared to those with shorter follow-up, underscoring the importance of sustained engagement to achieve durable benefits. Similarly, trials with high app adherence (≥75% of users) demonstrated significantly stronger effects (RR 0.39; 95% CI: 0.20–0.78; I^2^ = 26%), suggesting that user interaction is not merely a passive metric but a pivotal determinant of intervention efficacy. The TELE-ACS and WeChat HBCR studies, which incorporated clinician feedback and personalized monitoring, demonstrated the most pronounced effects, highlighting the value of bidirectional communication and individualized care pathways [11]. Although the included studies enrolled both elective and urgent PCI patients, no stratified analyses were performed. Available data suggest similar adherence across subgroups, but potential differences in engagement remain unexplored.

Beyond readmission rates, three of the four trials assessed patient-reported outcomes. Improvements in health-related quality of life, self-efficacy, and satisfaction with care were consistently reported in the trials that assessed these outcomes. These gains are particularly relevant for patients who may be unable to attend conventional cardiac rehabilitation due to logistical, financial, or geographic barriers [12,13]. As such, app-based care may not only reduce hospital utilization but also fill longstanding gaps in the delivery of comprehensive secondary prevention. This is especially pertinent in low-resource settings, where the burden of cardiovascular disease is rising and infrastructure for traditional follow-up is limited [14].

Looking ahead, these findings underscore the growing potential of digital health in transforming cardiovascular care. With rising smartphone penetration and increasing acceptance of telehealth modalities, future interventions can evolve toward greater personalization using artificial intelligence, integration with wearable sensors, and real-time data analytics [15,16]. The next generation of trials should focus on long-term outcomes, cost-effectiveness, and implementation in diverse healthcare systems. Importantly, digital tools should be co-designed with patients and clinicians to ensure usability, cultural relevance, and equity in access. If effectively scaled and integrated into standard care pathways, app-based interventions could redefine the post-MI recovery landscape by delivering consistent, patient-centered support at the population level.

## 5. Limitations

This meta-analysis has several limitations that should be acknowledged. First, only four randomized controlled trials met inclusion criteria, which limits the statistical power of subgroup analyses and the generalizability of the pooled estimates. Second, while all studies reported on unplanned hospital readmissions using consistent definitions, emergency department visits were not uniformly documented across trials. Although ED presentations were more frequent than readmissions in some studies, they were not reliably linked to hospitalization or adjudicated as cardiovascular-related and were therefore excluded from pooled analysis. This exclusion may underestimate the broader impact of digital interventions on acute care utilization. Third, MACEs were reported in only two trials and with heterogeneous definitions, precluding meaningful meta-analysis for this important secondary outcome. Fourth, intervention designs varied considerably in content, duration, and clinician involvement, contributing to moderate heterogeneity in the effect estimates. Fifth, the presence and type of heart failure among included patients was not uniformly documented, limiting assessment of intervention efficacy in this high-risk subgroup. Additionally, app adherence data were not consistently reported, limiting the ability to robustly assess dose–response relationships. Finally, none of the included studies provided formal cost-effectiveness evaluations or long-term sustainability data, and the small number of trials precluded formal assessment of publication bias (Table 4).

## 6. Future Directions

In light of the current findings and the limitations acknowledged, several future directions emerge. First, future randomized trials should aim to broaden inclusion criteria to capture more heterogeneous populations, particularly those with comorbidities, limited digital literacy, or socioeconomic barriers. This would enhance the external validity and relevance of digital interventions in real-world settings. Second, the development and application of standardized outcome definitions—especially for unplanned hospital readmissions, major adverse cardiovascular events (MACEs), and patient-reported quality of life—are essential. Such harmonization would facilitate cross-study comparisons, enable robust meta-analyses, and support clinical and regulatory decision-making. Third, cost-effectiveness remains a major gap. None of the included trials conducted formal economic evaluations, which are crucial for informing healthcare policy and reimbursement decisions. Implementation research should also assess practical barriers and enablers to adoption, particularly in resource-constrained healthcare systems where the potential for digital health’s impact may be greatest.

Fourth, greater granularity in the evaluation of app design is needed. Future research should explore which specific components—such as real-time clinician messaging, automated symptom monitoring, or behavioral reinforcement—are most effective in driving clinical benefit and sustaining engagement. Lastly, the long-term sustainability of digital engagement warrants further study. While short-term adherence was generally high, strategies to promote consistent usage beyond the initial months of follow-up remain underexplored. The integration of artificial intelligence and wearable technologies may facilitate more responsive, personalized interventions, potentially improving both adherence and outcomes. Collectively, these directions will be instrumental in transitioning mobile health applications from adjunctive tools to integral components of comprehensive, evidence-based secondary prevention in cardiovascular care.

## 7. Conclusions

Smartphone-based interventions for secondary prevention following myocardial infarction or PCI offer a promising tool for enhancing post-MI and post-PCI care. This meta-analysis shows consistent evidence that these digital tools can reduce unplanned hospital readmissions, particularly when interventions are sustained and user engagement is high. These findings support further integration of app-based strategies into routine cardiovascular follow-up. Future research should focus on standardizing outcomes, assessing cost-effectiveness, and leveraging evolving technologies to broaden their reach and impact.

## Figures and Tables

**Table 1:** Study characteristics.

Study	Year	Country	Sample Size (N)	Follow-Up Duration	Population	App Features	Control Group	Primary Outcomes	Secondary Outcomes
TELE-ACS	2022	United Kingdom	337	12 months	Post-MI/PCI	Education, reminders, clinician messaging, symptom tracking	Usual care without app	Unplanned readmissions	QoL, medication adherence, emergency department visits
afterAMI	2023	Poland	100	6 months	Post-MI	Daily notifications, secure messaging, medication reminders	Usual care without app	Unplanned readmissions, urgent visits and MACEs	QoL, satisfaction, adherence
ToDo-CR	2023	Australia	120	3 months	Post-PCI	Behavioral activation, step tracking, lifestyle coaching	Usual care without app	Unplanned readmissions	QoL, physical activity, behavior change
WeChat HBCR	2024	China	270	42 months	Post-MI/PCI	Cardiac rehab modules, peer support, real-time physician feedback	Usual care without app	MACEs, unscheduled readmission	QoL, physical activity, LDL-C, blood pressure

app, application; BP, blood pressure; CR, cardiac rehabilitation; LDL-C, low-density lipoprotein cholesterol; MACEs, major adverse cardiovascular events; MI, myocardial infarction; PCI, percutaneous coronary intervention; QoL, quality of life; RCT, randomized controlled trial.

**Table 2:** Baseline demographics of participants.

Study	Mean Age (Years)	Male (%)	HTN (%)	DM (%)	Smoking (%)	Hyperlipidemia (%)	Previous MI (%)	DAPT (%)	Statin Use (%)
TELE-ACS	61.4	68	76	25	21	85	29	94	98
afterAMI	58.3	62	63	12	17	72	22	96	97
ToDo-CR	60.2	65	58	22	42	61	24	91	94
WeChat HBCR	67.4	71	49	36	35	58	32	93	96

DAPT, dual antiplatelet therapy; DM, diabetes mellitus; HTN, hypertension; MI, myocardial infarction; PCI, percutaneous coronary intervention.

**Table 3:** Reported outcomes.

Study	Randomization Process	Deviations from Intended Interventions	Missing Outcome Data	Measurement of the Outcome	Selection of the Reported Result
TELE-ACS	Low risk—Computer-generated sequence and balanced groups	Low risk—Minimal deviation, high adherence, monitored use	Low risk—High retention and complete follow-up	Low risk—Objective outcomes (readmissions), possible blinding	Low risk—Trial registered, prespecified outcomes reported
afterAMI	Low risk—Adequate randomization and allocation concealment	Some concerns—Open-label with unclear handling of protocol deviations	Low risk—Follow-up > 95%, well-balanced	Low risk—Outcomes objectively verified	Low risk—Registered protocol followed
ToDo-CR	Low risk—Random sequence generation with no baseline imbalance	Low risk—Good adherence and protocol fidelity	Low risk—Attrition < 5%, similar between groups	High risk—Patient-reported outcomes without blinding	Low risk—No selective reporting identified
WeChat HBCR	Low risk—Randomization method adequately described	Low risk—No major deviations reported	High risk—>10% missing data, no imputation analysis	Low risk—Objective outcomes with automated logs and tracking	Low risk—Outcomes matched trial registration

**Table 4:** Detailed risk of bias assessment with justifications.

Study	Randomization Process	Deviations from Intended Interventions	Missing Outcome Data	Measurement of the Outcome	Selection of the Reported Result
TELE-ACS	Low risk—Computer-generated sequence and balanced groups	Low risk—Minimal deviation, high adherence, monitored use	Low risk—High retention and complete follow-up	Low risk—Objective outcomes (readmissions), possible blinding	Low risk—Trial registered, prespecified outcomes reported
afterAMI	Low risk—Adequate randomization and allocation concealment	Some concerns—Open-label with unclear handling of protocol deviations	Low risk—Follow-up > 95%, well-balanced	Low risk—Outcomes objectively verified	Low risk—Registered protocol followed
ToDo-CR	Low risk—Random sequence generation with no baseline imbalance	Low risk—Good adherence and protocol fidelity	Low risk—Attrition < 5%, similar between groups	High risk—Patient-reported outcomes without blinding	Low risk—No selective reporting identified
WeChat HBCR	Low risk—Randomization method adequately described	Low risk—No major deviations reported	High risk—>10% missing data, no imputation analysis	Low risk—Objective outcomes with automated logs and tracking	Low risk—Outcomes matched trial registration

ACS

Acute Coronary Syndrome

ARR

Absolute Risk Reduction

CI

Confidence Interval

ED

Emergency Department

HRQoL

Health-Related Quality of Life

LDL-C

Low-Density Lipoprotein Cholesterol

MACEs

Major Adverse Cardiovascular Events

MI

Myocardial Infarction

mHealth

Mobile Health

NNT

Number Needed to Treat

PCI

Percutaneous Coronary Intervention

QoL

Quality of Life

RCT

Randomized Controlled Trial

RR

Risk Ratio

## Data Availability

Data available upon request to the corresponding author.

## Abbreviations

The following abbreviations are used in this manuscript:

ACS	Acute Coronary Syndrome
ARR	Absolute Risk Reduction
CI	Confidence Interval
ED	Emergency Department
HRQoL	Health-Related Quality of Life
LDL-C	Low-Density Lipoprotein Cholesterol
MACEs	Major Adverse Cardiovascular Events
MI	Myocardial Infarction
mHealth	Mobile Health
NNT	Number Needed to Treat
PCI	Percutaneous Coronary Intervention
QoL	Quality of Life
RCT	Randomized Controlled Trial
RR	Risk Ratio
